# Antiarthritic potential of the butanol fraction of *Sesuvium sesuvioides*: An *in vitro*, *in vivo*, and *in silico* evaluation

**DOI:** 10.3389/fphar.2023.1136459

**Published:** 2023-05-26

**Authors:** Muhammad Sajid-ur-Rehman, Saiqa Ishtiaq, Hanan Y. Aati, Asmaa E. Sherif, Mohsin Abbas Khan, Mussadique Hussain, Muhammad Sohaib Khan, Maqsood Ahmed, Muhammad Jawad Naseem, Kashif-ur-Rehman Khan

**Affiliations:** ^1^ Department of Pharmacognosy, Faculty of Pharmacy, The Islamia University of Bahawalpur, Bahawalpur, Pakistan; ^2^ Department of Pharmacognosy, University of the Punjab, University College of Pharmacy, Lahore, Pakistan; ^3^ Department of Pharmacognosy, College of Pharmacy, King Saud University, Riyadh, Saudi Arabia; ^4^ Department of Pharmacognosy, College of Pharmacy, Prince Sattam Bin Abdulaziz University, Alkharj, Saudi Arabia; ^5^ Department of Pharmacognosy, Faculty of Pharmacy, Mansoura University, Mansoura, Egypt; ^6^ Department of Pharmaceutical Chemistry, Faculty of Pharmacy, The Islamia University of Bahawalpur, Bahawalpur, Pakistan; ^7^ Department of Pharmacology, Faculty of Pharmacy, The Islamia University of Bahawalpur, Bahawalpur, Pakistan; ^8^ College of Pharmacy and Integrated Research Institute for Drug Development, Dongguk University-Seoul, Goyang-si, Gyeonggi-do, Republic of Korea; ^9^ Division of Bioorganic Chemistry, School of Pharmacy, Saarland University, Saarbrueeken, Germany

**Keywords:** arthritis, *Sesuvium sesuvioides*, complete Freund’s adjuvant, inflammatory mediators, oxidative stress, molecular docking studies

## Abstract

*Sesuvium sesuvioides* (Fenzl) Verdc (Aizoaceae) has been traditionally used in the treatment of inflammation, arthritis, and gout. However, its antiarthritic potential has not been evaluated scientifically. The current study was designed to assess the antiarthritic properties of the *n*-butanol fraction of *S. sesuvioides* (SsBu) by phytochemical analysis, *in vitro* and *in vivo* pharmacological activities, and *in silico* studies. Phytochemical analysis showed total phenolic contents (90.7 ± 3.02 mg GAE/g) and total flavonoid contents (23.7 ± 0.69 mg RE/g), and further analysis by GC-MS identified possible bioactive phytocompounds belonging to phenols, flavonoids, steroids, and fatty acids. The *in vitro* antioxidant potential of SsBu was assessed by DPPH (175.5 ± 7.35 mg TE/g), ABTS (391.6 ± 17.1 mg TE/g), FRAP (418.2 ± 10.8 mg TE/g), CUPRAC (884.8 ± 7.97 mg TE/g), phosphomolybdenum (5.7 ± 0.33 mmol TE/g), and metal chelating activity (9.04 ± 0.58 mg EDTAE/g). Moreover, in the *in vitro* studies, inhibition (%) of egg albumin and bovine serum albumin denaturation assays showed that the anti-inflammatory effect of SsBu at the dose of 800 μg/ml was comparable to that of diclofenac sodium used as a standard drug. The *in vivo* antiarthritic activity was assessed to determine the curative impact of SsBu against formalin-induced (dose-dependent significant (*p* < 0.05) effect 72.2% inhibition at 750 mg/kg compared to standard; 69.1% inhibition) and complete Freund’s adjuvant-induced arthritis (40.8%; inhibition compared to standard, 42.3%). SsBu significantly controlled PGE-2 level compared to the control group (*p* < 0.001) and restored the hematological parameters in rheumatoid arthritis. Treatment with SsBu significantly reduced oxidative stress by reinstating superoxide dismutase, GSH, and malondialdehyde along with pro-inflammatory markers (IL-6 and TNF-α) in arthritic rats. Molecular docking revealed the antiarthritic role of major identified compounds. Kaempferol-3-rutinoside was found to be more potent for COX-1 (−9.2 kcal/mol) and COX-2 inhibition (−9.9 kcal/mol) than diclofenac sodium (COX-1, −8.0 and COX-2, −6.5 kcal/mol). Out of the 12 docked compounds, two for COX-1 and seven for COX-2 inhibition showed more potent binding than the standard drug. The results from the *in vitro*, *in vivo*, and *in silico* approaches finally concluded that the *n*-butanol fraction of *S. sesuvioides* had antioxidant and antiarthritic potential, which may be due to the presence of potential bioactive compounds.

## 1 Introduction

Rheumatoid arthritis (RA) is a common chronic auto-immune inflammatory disorder that commonly involves the joints of the hand, wrist, shoulder, and knees (Kamarudin et al., 2012). The pathological changes include infiltration of inflammatory cells and hyperplasia of the synovial membrane, which often progresses to a chronic state with functional disability (Patel et al., 2016). This syndrome also involves the secretion of pro-inflammatory cytokines, such as IL-6, IL-1, and TNF-α (Gupta et al., 2020). These cytokines trigger the synovial angiogenesis responsible for initiating pannus and cartilage damage and augmenting inflammatory cells and synovitis (Jian-Ping et al., 2018).

Non-steroidal anti-inflammatory drugs, steroids, disease-modifying antirheumatic drugs, and immunosuppressive drugs are commonly used to ameliorate inflammatory and rheumatic diseases (Pu et al., 2016), but they can cause severe side effects. Most of these drugs could result in gastrointestinal illnesses, cardiovascular problems, and reproductive toxicities (Peng et al., 2016). Therapy is intended to relieve symptomatic inflammation, reduce bone structure damage, improve joint function, and target systematic involvement (Saleem et al., 2020). In this way, an alternative therapy is needed to relieve rheumatoid disorders. The therapeutic effects of medicinal plants have drawn more attention among scientists for RA (Zhang et al., 2018). Phytoremedies exert beneficial effects not only on the disease symptoms but also on the pathogenesis of the disease (Jin et al., 2019). In Pakistan, 0.5% of the population is affected by RA (Sultana et al., 2020). The female population is more likely to deal with arthritis due to hereditary factors. In Pakistan, herbal remedies are more acceptable than conventional therapy as they are considered safe for the treatment of RA by most ethnic societies (Hasan et al., 2015).

The Cholistan Desert in Pakistan is rich in medicinal plants that can be used for treating rheumatoid arthritis or related disorders. *Leptadenia pyrotechnica*, *Cassia italica*, *Alhagi mourorum*, and *Salvadora oleoides* are commonly used in the treatment of arthritic diseases (Rehman et al., 2015). *Sesuvium sesuvioides* (Fenzl) Verdc (Aizoaceae) is an amazing herb found in the Cholistan Desert that has been used in the treatment of arthritis, inflammation, and gout by the local people (Ahmad et al., 2014; Sajid-Ur-Rehman et al., 2021). A number of medicinal plants and their isolated compounds have been used to relieve arthritis for thousands of years (Gupta et al., 2020). Many plant-based constituents possess significant therapeutic values, such as downregulation of inflammatory channels, and are potential candidates for the treatment of arthritis (Hariyadi and Sahu, 2020). The presence of a number of phytochemicals belonging to the phenols, flavonoids, glycosides, coumarins, terpenoids, saponins, and fatty acid esters has been reported in *S. sesuvioides* by GC-MS analysis (Sajid-Ur-Rehman et al., 2021). Phenols, flavonoids, steroids, coumarins, and terpenoids possess antioxidant, anti-inflammatory, and antiarthritic potential by inhibiting the release of pro-inflammatory cytokines and oxidative stress (Carvalho et al., 2019; Mohammed and Hassan, 2019; Direito et al., 2021; Malek Mahdavi et al., 2021; Yosri et al., 2022). The identification of these natural substances that protect the tissues from chronic inflammatory disorders would offer the opportunity to enhance antiarthritic therapy.

*Sesuvium sesuvioides* has been reported to reduce inflammation, pain, and fever in an animal model in a dose-dependent manner (Sajid-Ur-Rehman et al., 2021). However, there exists a gap in finding the mechanism of inflammatory disorders and arthritis. The present study aims to find the antiarthritic potential of *S. sesuvioides* by blocking pro-inflammatory mediators and to justify the folk uses scientifically.

## 2 Results

### 2.1 Total phenolic (TPC) and flavonoid contents (TFC) and antioxidant potential

The SsBu showed significant TPC (90.7 ± 3.02 mg GAE/g) and TFC values (23.7 ± 0.69 mg RE/g). The significant values of DPPH, ABTS, FRAP, CUPRAC, PBD, and metal chelating methods showed the antioxidant potential of SsBu, and the values are presented in Table 1.

**TABLE 1:** Total phenolic and flavonoids contents and antioxidant potential of SsBu.

Sample	TPC (mg GAE/g)	TFC (mg RE/g)	DPPH (mg TE/g)	ABTS (mg TE/g)	CUPRAC (mg TE/g)	FRAP (mg TE/g)	PBD (mmol TE/g)	Metal chelating (mg EDTAE/g)
SsBu	90.7 ± 3.02	23.7 ± 0.69	175.5 ± 7.35	391.6 ± 17.1	884.8 ± 7.97	418.2 ± 10.8	5.7 ± 0.33	9.04 ± 0.58

Data are represented as mean ± SEM. n = 3. GAE, gallic acid equivalents; RE, rutin equivalents; TE, Trolox equivalents; EDTA, ethylenediaminetetraacetic acid.

### 2.2 GC-MS analysis

**FIGURE 1:**
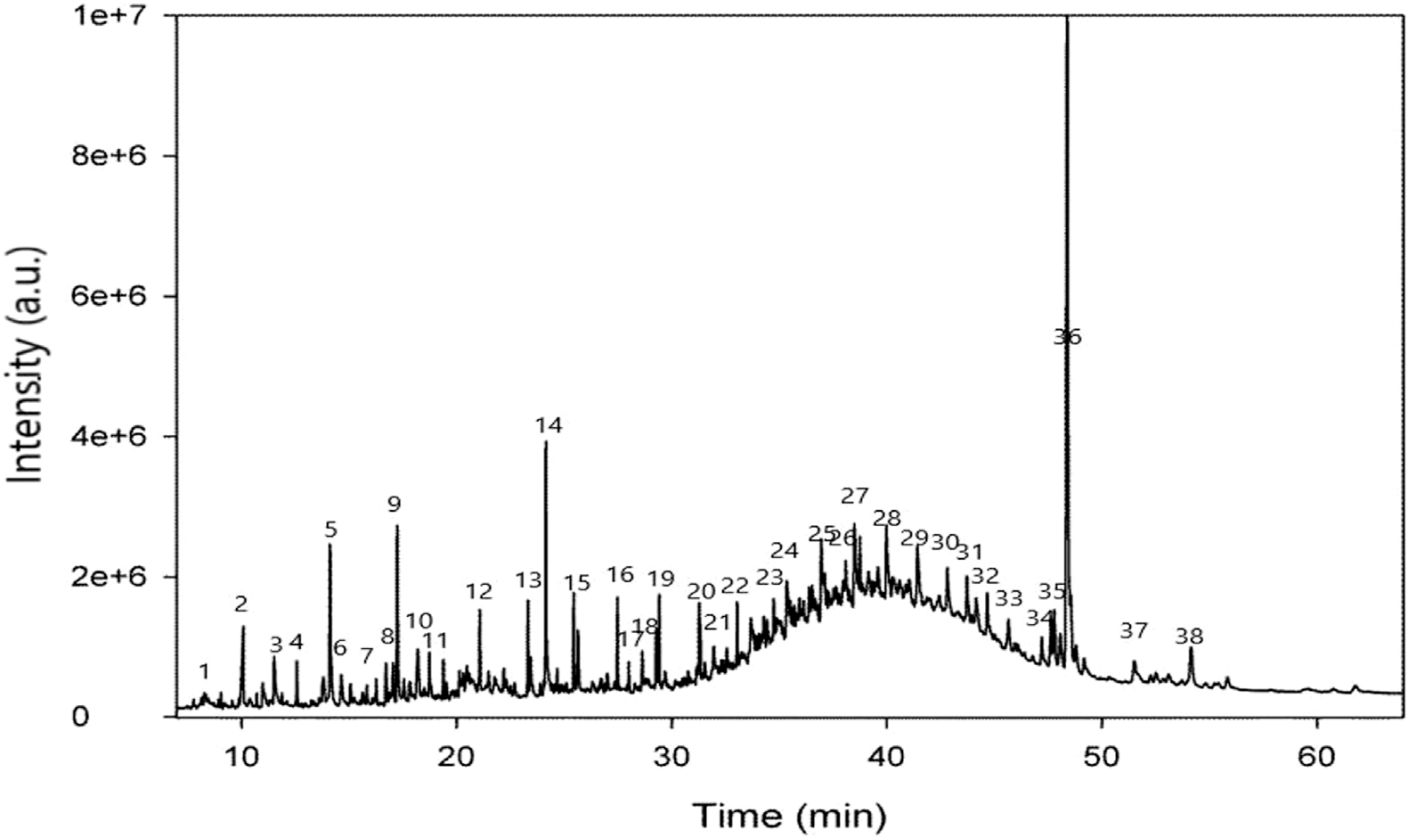
GC-MS chromatogram of SsBu.

Identification of secondary metabolite (Table 2 and Figure 1) was conducted by GC-MS analysis of the SsBu sample. The result of GC-MS analysis revealed 38 compounds belonging to various chemical classes of bioactive phytochemicals (phenols, flavonoids terpenoids, aldehydes, steroids, and fatty acids). These chemical classes were reported to show pharmacological activities by various researchers (Ahmed et al., 2022b; Dilshad et al., 2022; Ghalloo et al., 2022).

**TABLE 2:** Phytochemicals identified by GC-MS analysis.

Sr. no.	RT (min)	Compound name	Peak area %	Molecular mass (amu)	Molecular formula
1	7.78	5-Hydroxy-4-octanone	0.31	144.12	C_8_H_16_O_2_
2	9.98	Phenol, 2,2′-methylene-bis, 6-(1,1-dimethyl-ethyl)-4-methyl	2.49		C_23_H_32_O_2_
3	11.5	1-Heptadecene	1.61	238.45	C_17_H_34_
4	12.6	2-Methoxy-4-vinylphenol	1.49	150.07	C_9_H_10_O_2_
5	14.2	Bis (2-ethylhexyl) fumarate	4.21	340.56	C_20_H_36_O_4_
6	14.6	5,7-Dihydroxy-6,8-dimethoxy-2-phenyl-4H-chromen-4-one	1.05	314.08	C_17_H_14_O_6_
7	15.8	2-Methyl-4-chlorophenol	0.69	142.02	C_7_H_7_C_lO_
8	16.7	Methyl 13-methyl-tetradecanoate	0.91	256.40	C_16_H_36_O_2_
9	17.2	(z,z)-2-Methyl-3,13-octadecadienol	3.51	280.28	C_19_H_36_O
10	18.3	2,4-Di-tert-butyl-6-nitrophenol	1.35	251.15	C_14_H_21_NO_3_
11	19.4	2-(4a,8-Dimethyl-1,2,3,4,4a,5,6,7-octahydro-2-naphthalenyl)2-propen-1-ol	1.04	220.18	C_15_H_24_O
12	21.1	Octadec-9-enoic acid	1.66	282.50	C_18_H_34_O_2_
13	23.5	5,8-Dimethoxy-2H-chromen-2-one	1.76	206.06	C_11_H_10_O_4_
14	24.4	Phytol	5.41	296.31	C_20_H_40_O
15	25.7	Guaiacin	1.69	328.17	C_20_H_24_O_4_
16	27.1	4-(Hydroxymethyl)benzaldehyde	1.41	136.05	C_8_H_8_O_2_
17	28.1	2-Methyltetracosane	0.65	352.41	C_25_H_52_
18	28.8	6-Methoxy-2,2-dimethyl-2H-1-benzopyran	0.71	190.10	C_12_H_14_O_2_
19	29.4	Di (2-propyl-pentyl) phthalate	1.53	390.28	C_24_H_38_O_4_
20	31.3	3-Ethyl-5-methyl heptane	2.02	142.17	C_10_H_22_
21	31.8	4,4,5,8-Tetramethylchroman-2-ol	0.59	206.28	C_13_H_18_O_2_
22	32.2	Docosanoic acid, 8,9,13-trihydroxy, methyl ester	1.62	402.33	C_23_H_46_O_5_
23	34.6	7-Hydroxy-2H-chromen-2-one	0.77	162.1	C_9_H_6_O_3_
24	35.3	(3s)-4H-Pyran-4-one, 2,3 dihydro-3,5- dihydroxy-6- methyl-	1.82	144.04	C_6_H_8_O_4_
25	36.9	d-Camphoric acid	2.41	200.10	C_10_H_16_O_4_
26	37.9	3′,4′,5,7-Tetrahydroxy-3-methoxyflavone	1.21	316.06	C_16_H_12_O_7_
27	38.3	Isorhamnetin 3-O-glucoside	2.92	478.11	C_22_H_22_O_12_
28	40.2	4,8,12,16-Tetramethylheptadecan-4-olide	2.05	324.5	C_21_H_40_O_2_
29	41.4	Kaempferol-3-rutinoside	2.79	594.16	C_27_H_30_O_15_
30	42.8	5,7,2′-Trihydroxy-6-methoxy isoflavone	1.71	300.26	C_16_H_12_O_6_
31	43.7	2-(3,4-Dihydroxyphenyl) ethyl 3-O-(6-deoxy-beta-lamannopyranosyl)-6-O-(2E)-3-(3,4-dihydroxyphenyl) prop-2-enoyl]-beta-D-glucopyranoside	1.46	641.23	C_29_H_36_O_15_
32	44.6	3,4,5-Trimethoxyphenyl 6-O-thio-β-D-glucopyranoside	1.35	426.08	C_15_H_22_O_12_S
33	45.5	Thionerol	1.15	170.11	C_10_H_18_S
34	47.3	10,12-Hexadecadien-1-ol	1.25	238.23	C_16_H_30_O
35	47.7	(E)-9-Octadecenoic acid ethyl ester	1.79	310.29	C_20_H_38_O_2_
36	48.6	Squalene	23.5	410.39	C_30_H_50_
37	51.5	n-Heptadecanol-1	1.29	256	C_17_H_36_O
38	54.3	Stigmast-5-en-3-ol, (3,beta)-	1.31	414.39	C_29_H_50_O

RT, retention time.

### 2.3 Effect of SsBu on prostaglandin E-2

**FIGURE 2:**
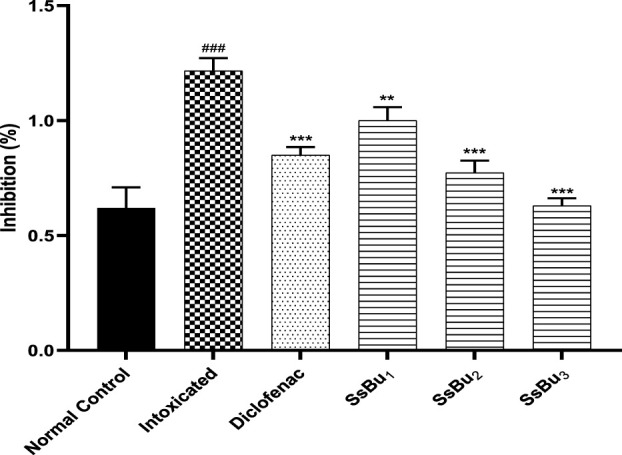
Effect of SsBu_1_, SsBu₂, and SsBu_3_ on the expression of prostaglandin E-2. One way ANOVA followed by Bonferroni's test was applied for statistical analysis. *p* < 0.001 (***), *p* < 0.01 (**), *p* < 0.05 (*) when the treatment group was compared to the intoxicated group (arthritic) and *p* < 0.001 (^###^) when the intoxicated was compared group to the control group.

When arthritic rats were treated with diclofenac sodium (standard drug) and SsBu_1_, SsBu_2_, and SsBu_3_, there was a significant (*p < 0.05*) decrease in the expression of PGE-2 in comparison with arthritic animals, as shown in Figure 2.

### 2.4 Effect of SsBu on inhibition (%) of egg albumin and bovine serum albumin denaturation

**FIGURE 3:**
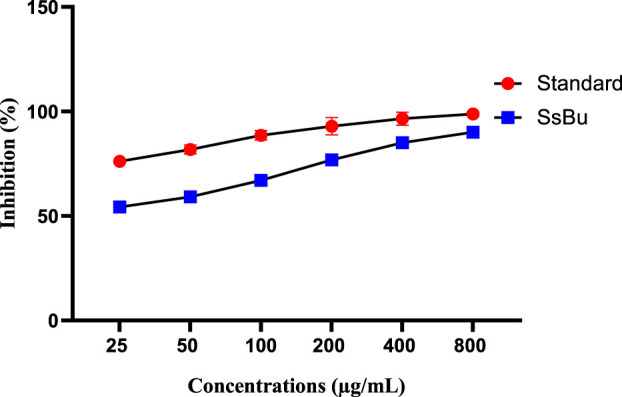
Effect of inhibition (%) of SsBu and standard drug (diclofenac sodium) on EA denaturation was evaluated at the end of the treatment using one-way ANOVA followed by Bonferroni’s test. Results are represented as the mean ± SEM for n = 3.

**FIGURE 4:**
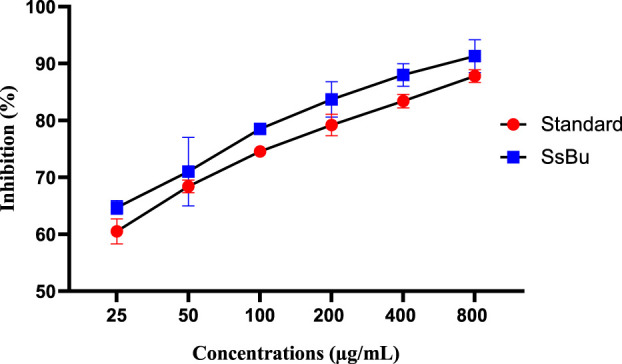
Effect of inhibition (%) of SsBu and standard drug (diclofenac sodium) on BSA denaturation. Results were evaluated at the end of the treatment using one-way ANOVA followed by Bonferroni’s test. Results are represented as the mean ± SEM for n = 3.

SsBu (90.06 ± 1.12) revealed significant (*p* < 0.05) inhibition of egg albumin denaturation at 800 μg/ml when compared with the standard drug (98.8 ± 0.51). SsBu (91.3 ± 1.67) showed highly significant inhibition (*p* < 0.001) of BSA denaturation at 800 μg/ml when compared with the standard drug (87.8 ± 0.63). The results are presented in Figures 3, 4.

### 2.5 Effect of SsBu on formalin-induced arthritis

SsBu_3_ significantly (*p* < 0.05) decreased paw diameter (72.2 %) in the treatment group compared with the standard group (69.1 %) on day 10. A description of the results is given in Table 3.

**TABLE 3:** Effect of SsBu on paw diameter (mm) of formalin-induced arthritis.

Treatment group	Day 0	Day 2	Day 4	Day 6	Day 8	Day 10
Normal control	3.87 ± 0.11	3.87 ± 0.11	3.86 ± 0.11	3.86 ± 0.11	3.86 ± 0.11	3.86 ± 0.11
Intoxicated	4.07 ± 0.11	5.8 ± 0.21^###^	7.27 ± 0.18^###^	9.29 ± 0.43^###^	12.77 ± 0.27^###^	15.16 ± 0.4^###^
Standard	4.1 ± 0.13	5.3 ± 0.14	6.13 ± 0.09^**^	5.87 ± 0.16^***^	5.25 ± 0.16^***^	4.68 ± 0.16^***^ (69.1%)
SsBu_1_	4.39 ± 0.11	5.85 ± 0.04	6.56 ± 0.08	6.35 ± 0.05^***^	6.09 ± 0.04^***^	5.9 ± 0.03 (61%)
SsBu_2_	4.13 ± 0.13	5.89 ± 0.07	6.54 ± 0.08	6.27 ± 0.16^***^	5.88 ± 0.17^***^	5.68 ± 0.17 (62.5%)
SsBu_3_	3.92 ± 0.15	5.9 ± 0.07	5.54 ± 0.09^***^	4.98 ± 0.11^***^	4.46 ± 0.13^***^	4.2 ± 0.13 (72.2%)

Data are represented as mean ± SEM; n = 6.

### 2.6 CFA-induced arthritis

**FIGURE 5:**
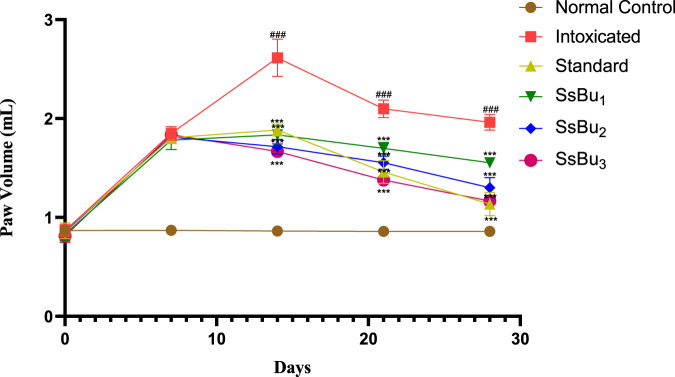
Bar graph representing the effect of SsBu_1_, SsBu_2_, and SsBu_3_ and standard drug on paw volume of CFA-induced arthritic rats. Results were evaluated at the end of the treatment using one-way ANOVA followed by Bonferroni’s test. Each point in the bar graph represents the mean ± SEM for n = 6 experiments on rats. *p* < 0.001 (***), *p* < 0.01 (**), *p* < 0.05 (*), when the treatment group was compared to the intoxicated group.

All CFA-induced arthritic animals presented chronic polyarthritic edema, beginning around day 7 and reaching its peak 14 days after injection. Throughout the 28-day experiment, there was no change in paw edema in the control group, while the standard diclofenac sodium significantly inhibited edema by 42.3%. Similarly, SsBu_1_, SsBu_2_, and SsBu_3_ showed 20.9%, 33.6%, and 40.9% inhibition, respectively. Table 4 shows the resultant values of this assay and graphical representation of results is given in Figure 5.

**TABLE 4:** Effect of SsBu on paw edema in CFA-induced arthritic rats.

Treatment group	Day 0	Day 7	Day 14	Day 21	Day 28
Normal control	0.86 ± 0.02	0.87 ± 0.01	0.86 ± 0.02	0.85 ± 0.02	0.85 ± 0.02
Intoxicated	0.86 ± 0.02	1.85 ± 0.02	2.61 ± 0.07^###^	2.09 ± 0.03^###^	1.96 ± 0.03^###^
Standard	0.86 ± 0.03	1.8 ± 0.02	1.88 ± 0.02***	1.46 ± 0.04^***^	1.13 ± 0.04^***^ (42.3%)
SsBu_1_	0.83 ± 0.02	1.78 ± 0.03	1.83 ± 0.03***	1.69 ± 0.02^***^	1.55 ± 0.01^***^ (20.9%)
SsBu_2_	0.85 ± 0.02	1.81 ± 0.02	1.71 ± 0.03***	1.55 ± 0.02^***^	1.3 ± 0.04^***^ (33.6%)
SsBu_3_	0.81 ± 0.02	1.83 ± 0.02	1.66 ± 0.02^***^	1.37 ± 0.01^***^	1.16 ± 0.02^***^ (40.8%)

Data are expressed as mean ± SEM; n = 6.

### 2.7 Effect of SsBu on hematological parameters

In the hematological parameters, SsBu significantly (*p < 0.05*) boosted the levels of Hb (13 ± 0.28 g/dl) and RBCs (6.84 ± 0.04 10^6^/µl), which were found in low levels during arthritis. Similarly, the total count of WBCs (6.26 ± 0.06 10^3^/µl), platelets (424 ± 7.79 10^3^/µl), and ESR (9.6 ± 0.02 mm/first hr) significantly reduced towards normal levels in arthritic animals treated with SsBu. At the same time, SsBu and the standard group showed a negative RF value, while the intoxicated group presented a positive RF value.

### 2.8 Histopathological judgments

**FIGURE 6:**
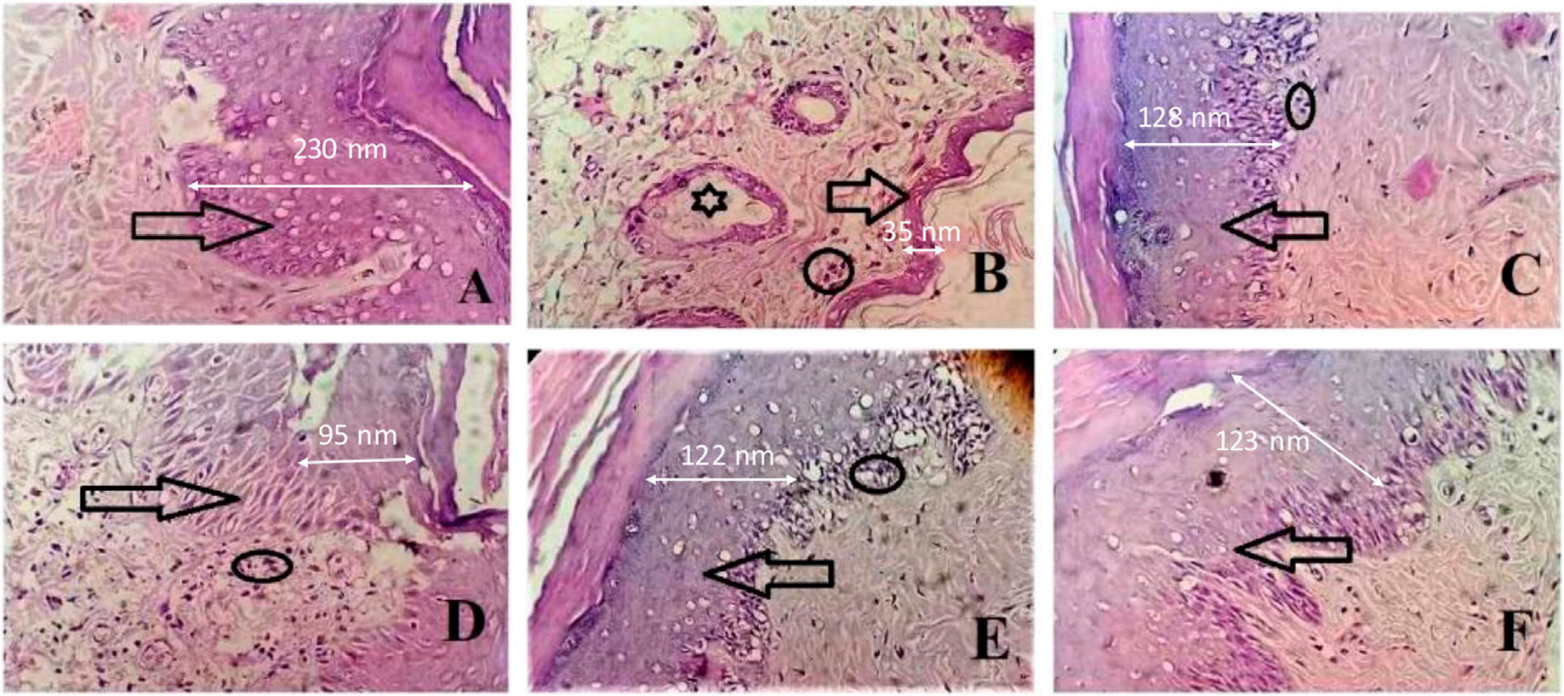
Effect of SsBu on histopathological findings from the CFA-induced arthritis rat model. **(A)** Normal control; arrow shows the normal epithelial layer of paw tissues; **(B)** intoxicated group indicating the markedly reduced epithelial layer of paw tissues (arrow), inflammatory cells (circle), and pannus formation (asterisk); **(C)** standard group; and **(D)** SsBu_1_-treated; **(E)** SsBu_2_-treated; and **(F)** SsBu_3_-treated groups. Dotted arrow shows the scale bar for the epithelial layer (2.5 cm length of the arrow equals to 100 nm).

From the histopathological judgments, it was noted that inflammatory cell infiltration, destruction of the epithelial cell of joint tissues, and pannus formation were observed at high levels in the CFA-induced arthritic group as presented in Figures 6A–F. SsBu_1_, SsBu_2_, and SsBu_3_ presented a reduction in inflammatory cell infiltration, destruction of the epithelial cell of joint tissues, and pannus formation. The antiarthritic potential of SsBu_3_ is more pronounced by observing the revival of normal cells along with the decline in inflammatory cell infiltration and pannus development. The control group lacked any inflammation and pannus growth. Moreover, the standard group showed a reduction in arthritic indices with fewer inflammatory cells.

### 2.9 Effect of SsBu on the levels of oxidative stress markers

The effect of SsBu on the levels of oxidative stress markers was determined according to the protocol described in ELISA kits.

#### 2.9.1 Estimation of SOD

**FIGURE 7:**
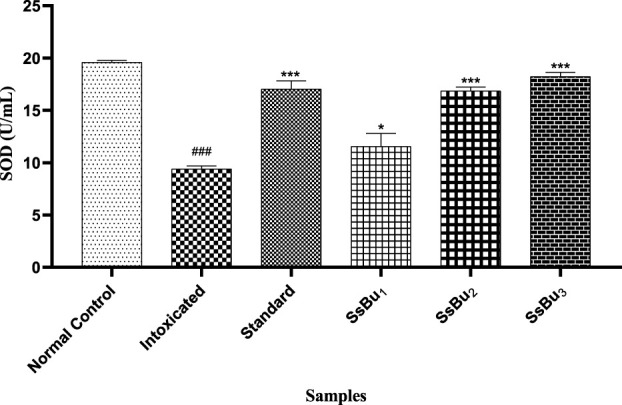
Bar graph representing the effect of treatment on SOD levels. The level of SOD was estimated at the end of the treatment using one-way ANOVA followed by Bonferroni’s test. Each point in the bar graph represents the mean ± SEM for n = 3 experiments on rats. *p* < 0.001 (***), *p* < 0.01 (**), *p* < 0.05 (*) when the treatment group was compared to the intoxicated group and *p* < 0.001 (^###^) when the intoxicated group was compared to the control group.

The SOD level of the intoxicated group (9.4 ± 0.16 U/ml) significantly declined (^###^ *p* < 0.001) in comparison with that of the control group (19.59 ± 0.11 U/ml). Standard (17.04 ± 0.44 U/ml) and SsBu (18.22 ± 0.23 U/ml) groups showed a statistical significance (^*^ *p* < 0.05) when compared to the intoxicated group as presented in Figure 7.

#### 2.9.2 Estimation of GSH

**FIGURE 8:**
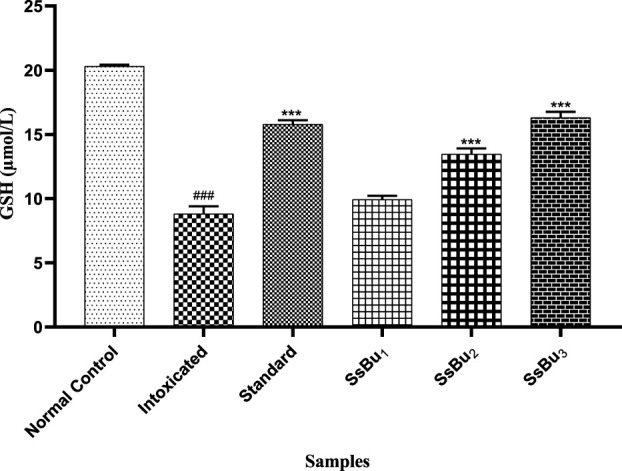
Bar graph representing the effect of treatment on GSH levels. The level of GSH was estimated at the end of the treatment using one-way ANOVA followed by Bonferroni’s test. Each point in the bar graph represents the mean ± SEM for n = 6 experiments on rats. *p* < 0.001 (***), *p* < 0.01 (**), *p* < 0.05 (*) when the treatment group was compared to the intoxicated group and *p* < 0.001 (^###^) when the intoxicated group was compared to the control group.

The level of GSH significantly declined (^###^ *p* < 0.001) in the plasma of the intoxicated group (8.8 ± 0.34) in comparison with the control group (20.31 ± 0.06). Standard (15.78 ± 0.18) and SsBu (16.30 ± 0.25) groups showed a statistical significance (^*^ *p* < 0.05) when compared to the intoxicated group as presented in Figure 8.

#### 2.9.3 Estimation of MDA

**FIGURE 9:**
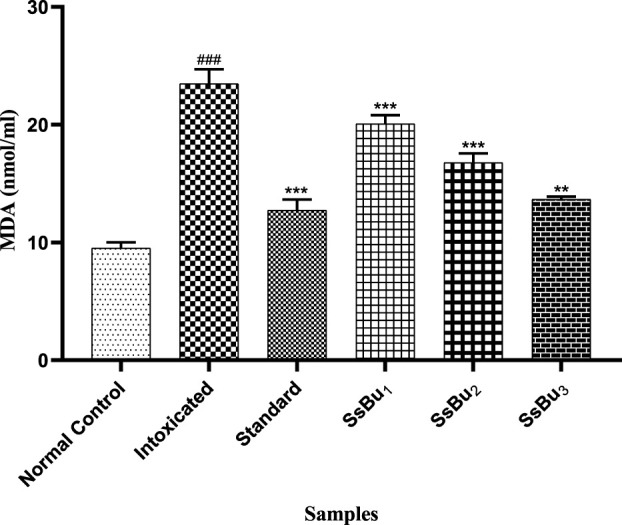
Bar graph representing the effect of treatment on MDA levels. The level of MDA was estimated at the end of the treatment using one-way ANOVA followed by Bonferroni’s test. Each point in the bar graph represents the mean ± SEM for n = 6 experiments on rats. *p* < 0.001 (***), *p* < 0.01 (**), *p* < 0.05 (*) when the treatment group was compared to the intoxicated group and *p* < 0.001 (^###^) when the intoxicated group was compared to the control group.

The level of MDA was significantly raised (^###^ *p* < 0.001) in the plasma of the intoxicated group (23.47 ± 0.71) in comparison with the control group (9.51 ± 0.28). Standard (12.72 ± 0.53) and SsBu (13.66 ± 0.13) groups showed a significance (^*^ *p* < 0.05) when compared to the intoxicated group as presented in Figure 9.

### 2.10 Effect of SsBu on the levels of pro-inflammatory cytokines

The effect of SsBu on the levels of pro-inflammatory cytokines, i.e., interleukin-6 (IL-6) and TNF-α, was determined according to the manufacturer’s instructions provided with the of ELISA kits.

#### 2.10.1 Estimation of the IL-6 level

**FIGURE 10:**
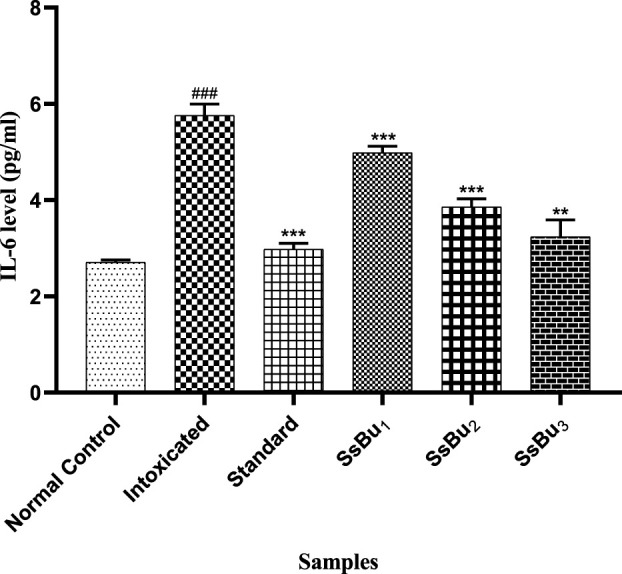
Bar graph representing the effect of SsBu on IL-6 levels. The level of IL-6 was estimated at the end of the treatment using one-way ANOVA followed by Bonferroni’s test. Each point in the bar graph represents the mean ± SEM for n = 6 experiments on rats. *p* < 0.001 (***), *p* < 0.01 (**), *p* < 0.05 (*) when the treatment group was compared to the intoxicated group and *p* < 0.001 (^###^) when the intoxicated group was compared to the control group.

The IL-6 level significantly increased (^###^ *p* < 0.001) in the intoxicated group (5.76 ± 0.13) when compared with the control group (2.7 ± 0.02). Standard (2.97 ± 0.07) and SsBu (3.23 ± 0.2) groups showed a significance (^*^ *p* < 0.05) when compared to the intoxicated group as presented in Figure 10.

#### 2.10.2 Estimation of TNF-α

**FIGURE 11:**
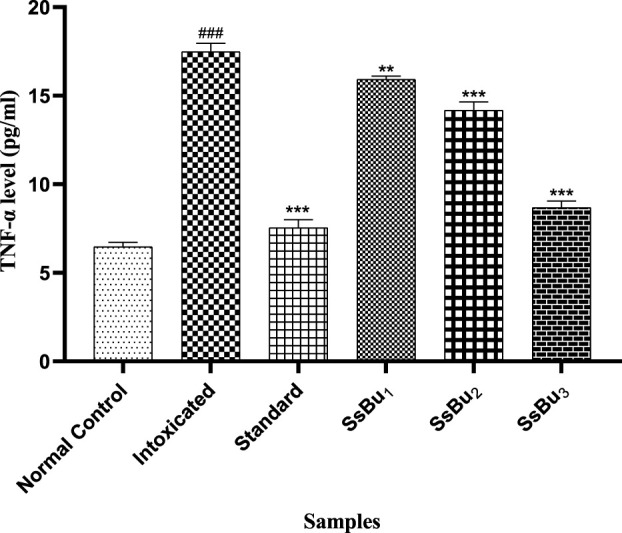
Bar graph representing the effect of SsBu on TNF-α levels. The level of TNF-α was estimated at the end of the treatment using one-way ANOVA followed by Bonferroni’s test. Each point in the bar graph represents the mean ± SEM for n = 6 experiments on rats. *p* < 0.001 (***), *p* < 0.01 (**), *p* < 0.05 *() when the treatment group was compared to the intoxicated group and *p* < 0.001 (^###^) when the intoxicated group was compared to the control group.

The TNF-α level significantly increased (^###^ *p* < 0.001) in the intoxicated group (17.48 ± 0.28) when compared with the control group (6.46 ± 0.14). Standard (7.54 ± 0.26) and SsBu (8.68 ± 0.2) groups showed a significance (^*^ *p* < 0.05) when compared to the intoxicated group as presented in Figure 11.

### 2.11 Molecular docking studies

**FIGURE 12:**
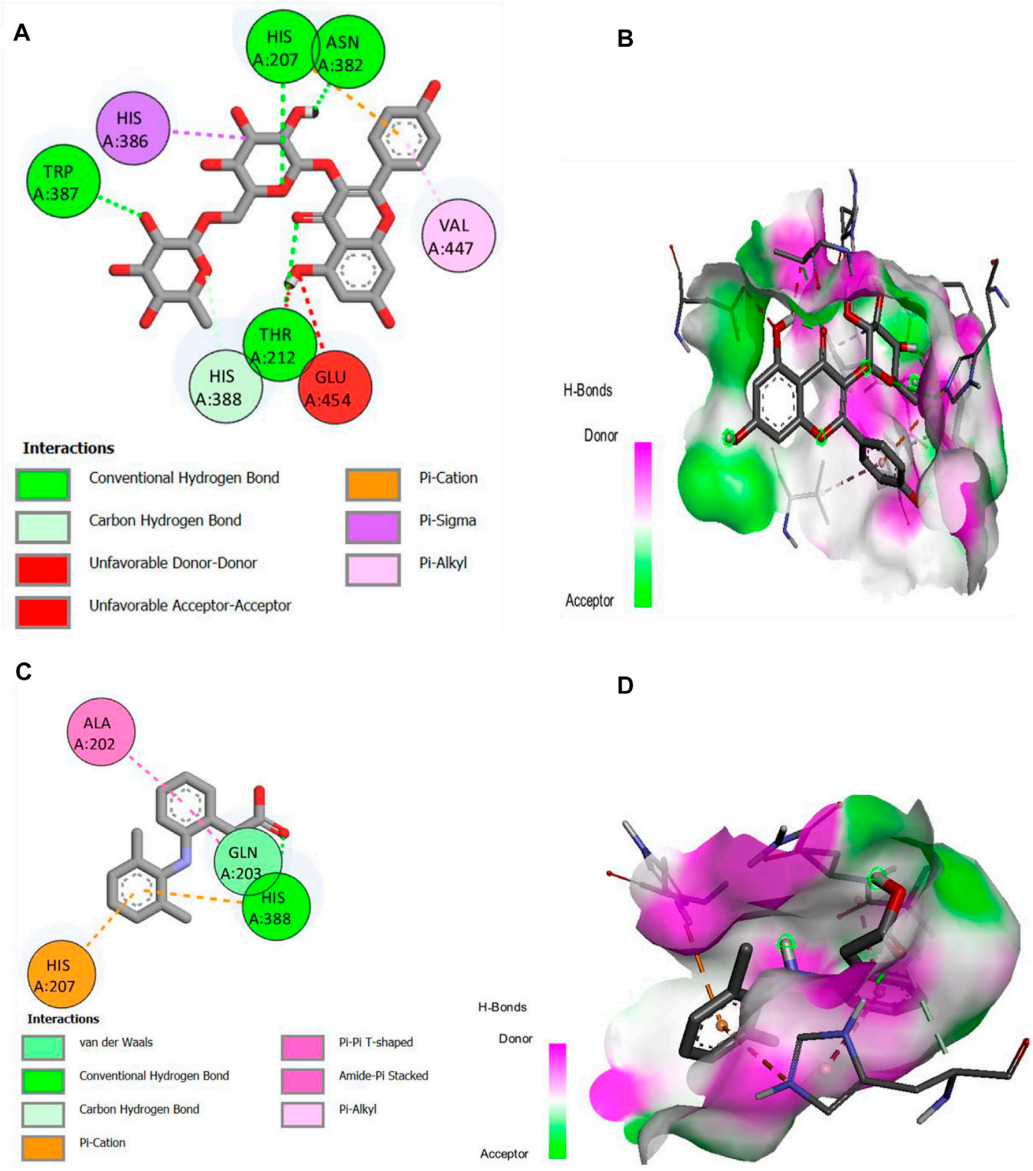
**(A,B)** 2D and 3D representation of binding interactions of kaempferol-3-rutinoside (ligand with the highest binding score) with COX-1 active site residues; **(C,D)** 2D and 3D representation of binding interactions of diclofenac sodium (standard drug).

**FIGURE 13:**
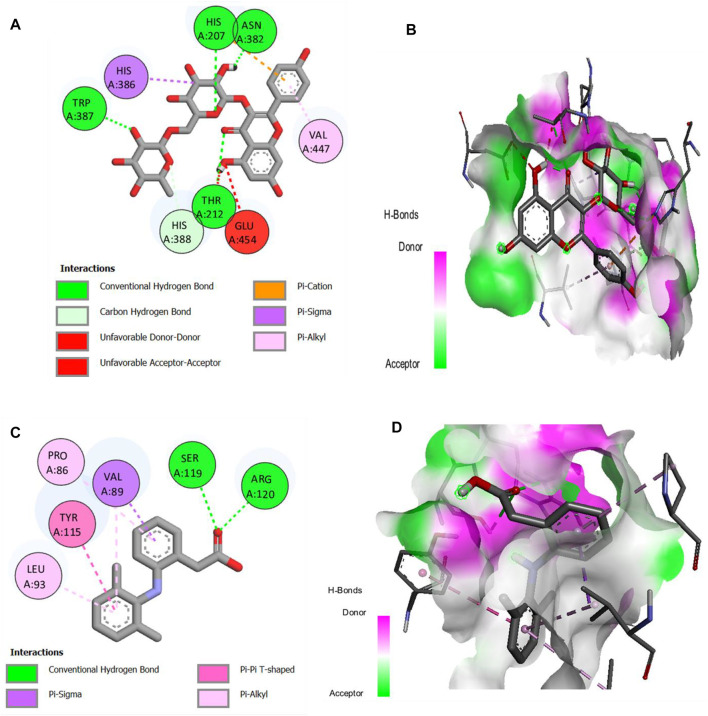
**(A,B)** 2D and 3D representation of binding interactions of kaempferol-3-rutinoside (ligand with the highest binding score) with COX-2 active site residues; **(C,D)** 2D and 3D representation of binding interactions of diclofenac sodium (standard drug).

The major compounds identified by GC-MS were docked against COX-1 and COX-2. The binding energies were calculated and are shown in Table 5, and some receptor–ligand interactions are shown in Figures 12, 13. A high binding affinity was observed for two out of the 12 docked compounds against COX-1 enzyme compared to the standard drug diclofenac sodium. The typical drug showed a binding energy of −8.0 kcal/mol, while kaempferol-3-rutinoside showed −9.2 kcal/mol and phenol, 2,2′-methylene-bis, 6-(1,1-dimethyl-ethyl)-4-methyl showed −8.2 kcal/mol. It is evident from the results that these two compounds may contribute to the main role of SsBu in anti-inflammatory and antiarthritic activities. Moreover, docking against the COX-2 enzyme showed that seven compounds, namely, squalene, phytol, bis (2-ethylhexyl) fumarate, isorhamnetin 3-O-glucoside, kaempferol-3-rutinoside, phenol, 2,2′-methylene-bis, 6-(1,1-dimethyl-ethyl)-4-methyl, and d-camphoric acid, have lower binding energies (−6.6 kcal/mol –9.9 kcal/mol) than diclofenac sodium (−6.5 kcal/mol), while (E)-9-octadecenoic acid ethyl ester exhibited equal binding energy (−6.5 kcal/mol) to the standard drug. Only four out of the 12 docked compounds showed a lower affinity with the COX-2 binding site compared to the standard (due to higher binding energies than the standard).

**TABLE 5:** Molecular docking study of selected ligands with COX-1 and COX-2 enzymes.

Sr. no.	Name of the docked ligand	Docking score with COX-1 (kcal/mol)	Docking score with COX-2 (kcal/mol)
1	Squalene	−7.0	−6.9
2	Phytol	−6.4	−6.9
3	Bis (2-ethylhexyl) fumarate	−6.7	−7.3
4	(z,z)-2-Methyl-3,13-octadecadienol	−5.7	−4.8
5	Isorhamnetin 3-O-glucoside	−7.9	−8.5
6	Kaempferol-3-rutinoside	−9.2	−9.9
7	Phenol, 2.2′-methylene-bis, 6-(1,1-dimethyl-ethyl)-4-methyl	−8.2	−7.2
8	d-Camphoric acid	6.7	−6.6
9	4.8,12,16-Tetramethylheptadecan-4-olide	−7.3	−6.4
10	3-Ethyl-5-methyl heptane	−4.7	−5.8
11	(3s)-4H-Pyran-4-one, 2,3 dihydro-3.5- dihydroxy-6- methyl-	−6.0	−5.9
12	(E)-9-Octadecenoic acid ethyl ester	−6.5	−6.5
13	Diclofenac sodium	−8	−6.5

## 3 Discussion

Arthritis is a multifactorial auto-immune disease with symptoms of chronic swelling in multiple joints with inflammatory cells and is often treated with NSAIDs as monotherapy or combinational therapy. Still, their serious side effects pose a challenge for better and safe remedies (Gupta et al., 2020). Medicinal plants have been used clinically to treat arthralgia, which is considered more tolerated and convenient for arthritic patients (Zhang et al., 2018).

The result of the present study demonstrated the antiarthritic properties of the butanol fraction of *S. sesuvioides* and the possible molecular and cellular mechanism of action. Prostaglandins usually relax arteriolar muscle cells and improve blood flow, thus considerably amplifying the exudates. CFA induction elevates PGE-2 levels and is responsible for increased paw edema volume in arthritic rats (Alzarea et al., 2022). SsBu significantly reduced the elevated PGE-2 level, suggesting a response of SsBu against CFA-induced inflammation and arthritis.

The effect of SsBu on elevated PGE-2 levels might be due to the presence of various phenolic and flavonoid compounds reported in GC-MS analysis. 2-Methoxy-4-vinylphenol, kaempferol-3-rutinoside, isorhamnetin 3-O-glucoside, and 6,7,3′-trihydroxy-5,2′-dimethoxy isoflavone are reported to inhibit the prostaglandins that might be responsible for the antiarthritic activity. SsBu presented significant inhibition of protein denaturation of egg and bovine serum albumin in *in vitro* analysis. Denaturation of protein cells and tissue injury are considered to be well correlated with inflammatory diseases and rheumatoid arthritis (Elisha et al., 2016; Osman et al., 2016). The inhibition of denaturation of proteins by SsBu supports and signifies apparent potential as an antiarthritic agent.

It has been reported that formalin-induced arthritis in animal models is used for the preclinical screening of NSAIDs and plant extract for antiarthritic potential (Fatima and Fatima, 2016). Formaldehyde degrades protein at the site of injection, which promotes the secretion of neurogenic histamine and aggravation of the immunological response to protect protein denaturation. Formaldehyde (0.1 ml, 5% v/v) produces inflammation of joints by denaturing proteins and thus induces immunological reactions against degraded products. Formaldehyde-induced biphasic arthritis initially produces pain, followed by the discharge of inflammatory cytokines, such as serotonin, bradykinin, and PGs (Saleem et al., 2020).

In this study, SsBu_1_, SsBu_2,_ and SsBu_3_ along with the standard drug showed a significant reduction in rat paw diameter on day 10. There was a significantly decreasing trend in the paw diameter of the diclofenac sodium-treated group. Similarly, paw diameter was significantly reduced in the SsBu-treated group. Several studies proved the antioxidant, anti-inflammatory, and antiarthritic potential of phenolic acids and flavonoids (dos Santos et al., 2020). Findings related to quantitative phytochemical analysis exposed the presence of phenols and flavonoids in SsBu, suggesting that the presence of phenols and flavonoids helped in reducing inflammation in arthritis.

CFA-induced arthritis was a recommended model because previous studies revealed that RA pathogenesis in humans and animals seems to be similar (Ahsan et al., 2021). Measurement of paw volume of CFA-induced arthritic and treated rats was assessed using a plethysmometer on days 7, 14, 21, and 28. The pathogenesis of CFA-induced arthritis includes the release of inflammatory cytokines, such as TNF-α, IL-1, IL-6, prostaglandins, and serotonins, that migrate to the affected area and lead to edema in particular tissues, such as joint capsule and ligaments (Kshirsagar et al., 2014; Nasuti et al., 2019). Inflammatory cytokines elicit the development of cellular immune response and T-cell activation. TNF-α is also involved in the continuation of inflammatory cascades, leading to the contraction of connective tissues. This flare in the injected paw is an immunological event, and a drug’s suppression of such lesions/irritant effect shows its immunosuppressant potential. In the intoxicated (arthritic) group, paw edema was sustained due to cellular infiltration, and, in the SsBu-treated group, paw edema was significantly reduced in contrast to the intoxicated group. The existence of these polyphenols in SsBu suggested its anti-inflammatory property, which is directly involved in the recovery of CFA-induced arthritis shown in the reduction of paw edema.

GC-MS analysis of the butanol fraction of *S. sesuvioides* identified several phytocompounds, such as methyl 13-methyl-tetradecanoate (antioxidant), phytol (anti-inflammatory and antiarthritic), 3′,4′,5,7-tetrahydroxy-3-methoxyflavone (antioxidant), 3,5-dihydroxy-6-methyl-2,3-dihydropyran-4-one (antioxidant), 4,4,5,8-tetramethylchroman-2-ol (anti-inflammatory), kaempferol-3-rutinoside (antioxidant and anticancer), 3,4,5-trimethoxyphenyl 6-O-sulfo-beta-D-glucopyranoside (anti-inflammatory), (E)-9-octadecenoic acid ethyl ester (anti-inflammatory), squalene (antioxidant), n-heptadecanol-1 (antiarthritic), and stigmast-5-en-3-ol, (3,beta) (antiarthritic and anti-inflammatory) (Pertuit et al., 2015; Sunita et al., 2017; Beulah et al., 2018; Lozano-Grande et al., 2018; Khanal and Patil, 2019; Carvalho et al., 2020; Razak et al., 2020; Li et al., 2021; Aati et al., 2022; Xie et al., 2022). A lot of phytocompounds have been reported to treat several auto-immune ailments, including arthritis, which has been evaluated in *in vivo* experiments (Sivasakthi et al., 2021). The existence of these phytocompounds in SsBu possessing antioxidant, anti-inflammatory, and antiarthritic potential might be responsible for reducing CFA-induced arthritis, which is evident by observing the decline in paw volume (swelling) in the rats. These phytocompounds are reported to downregulate NF-kB and reduce oxidative stress in joint tissues. They also decrease or inhibit the movement of inflammatory cells in the synovial tissues, thus reducing hyperalgesia in CFA-induced arthritis.

There is an increase in plasma levels of RF, WBCs, and platelets, while Hb and RBC levels decrease in CFA-induced arthritis (Table 6). The rise in the WBC count and RF level in arthritic conditions was reported to be due to a rise in the IL-1B level in the respective colony-stimulating factor. SsBu treatment tends to normalize WBC and RF levels. In addition, alterations in hematological parameters, such as Hb, RBC, and ESR, were also observed during arthritis. Hb, RBC, and ESR levels were reduced towards normal levels by the use of SsBu, which further supports its antiarthritic effect. The histopathological evaluation demonstrated that SsBu could effectively control the infiltration of inflammatory cells, mitigate the synovial hyperplasia, and protect bone destruction by RA, as shown in Figures 6D–F. These results authenticated the good therapeutic potential of SsBu on RA, which might be associated with the inhibition of TNF-α and IL-6 (Zhang et al., 2018).

**TABLE 6:** Effect of SsBu on hematological parameters.

Treatment group	Hb (g/dl)	RBCs (10^6^/µl)	WBCs (10^3^/µl)	Platelets (10^3^/µl)	ESR (mm/first hr)	RF (IU/ml)
Normal control	14 ± 0.02	7.28 ± 0.12	5.44 ± 0.04	323 ± 12.7	3.6 ± 0.28	15.2 ± 0.08 (-ve)
Intoxicated	8.7 ± 0.13	4.53 ± 0.04	9.8 ± 0.07	1,176 ± 6.92	22.8 ± 0.62	50.8 ± 0.63 (+ve)
Standard	12.1 ± 0.26	6.33 ± 0.04	7.8 ± 0.05	727 ± 8.6	12.2 ± 0.15	25.6 ± 0.4 (-ve)
SsBu_1_	9.19 ± 0.17	6.15 ± 0.28	8.61 ± 0.22	908 ± 9.84	17.7 ± 0.43	38.6 ± 0.64 (-ve)
SsBu_2_	12.13 ± 0.2	6.55 ± 0.03	7.33 ± 0.05	522 ± 6.92	12.8 ± 0.51	27.1 ± 0.62 (-ve)
SsBu_3_	13 ± 0.28	6.84 ± 0.04	6.26 ± 0.06	424 ± 7.79	9.6 ± 0.02	22.54 ± 0.6 (-ve)

Data are expressed as mean ± SEM; n = 3.

From the literature review, it has been reported that oxidative stress is involved in the pathology of RA, which was evident in the reduced levels of enzymatic antioxidants, such as SOD and GSH, and increased levels of MDA (Sukketsiri et al., 2016). Levels of SOD and GSH were maintained at near-normal levels through the oral administration of SsBu, which was found to be significant when compared to the arthritic animals. A similar increase in the level of SOD was also observed, and this increase in SOD and GSH enzymes appeared to be a protective effect against the intracellular oxygen-free radical (Suseela et al., 2021). Treatment with SsBu suppressed the rise in MDA, indicating a protective effect, and restored the altered indicator toward a normal level.

Inflammatory cytokines release, particularly IL-1β and TNF-α, have an esse cartilage in different impediments of inflammatory disorders and RA through the destruction of cartilages. TNF-α upregulates the expression of adhesion factor in synovial tissues and induces an inflammatory response. Similarly, IL-1β extends the expression of chemokines that induce the chemotaxis of neutrophils, resulting in pannus formation and the production of PGE-2. In this present study, SsBu significantly downregulated the levels of IL-1β and TNF-α compared to the arthritic animals. The aforementioned findings reveal that SsBu possesses potential antiarthritic properties. The underlying mechanism for the beneficial effect of SsBu could be mediated through several factors, such as regulation of anti-inflammatory cytokines, alleviating oxidative stress, and arthritic protective activities.

Molecular docking is a critical technique in computer-aided drug design for describing and explaining the interaction of ligand and receptor molecules that interact together in three-dimensional spaces. The lower the binding energies of ligand molecules with the receptor site of protein molecules, the higher the binding affinities and the more stable the receptor–ligand complex will be. Cyclooxygenases-1 and -2 are the key enzymes contributing to inflammation and pain related to arthritis. Therefore, the inhibition of these critical enzymes by bioactive molecules correlates with their efficacy in relieving the symptoms of arthritis. The docking results of identified compounds from SsBu showed that two compounds inhibited COX-1 more than the standard drug and may contribute to the plant’s role in treating arthritis. Moreover, COX-2 was inhibited by the majority of compounds with higher binding affinity compared to the standard drug, and one compound showed equal inhibition to diclofenac sodium. It substantiates that SsBu possesses a greater ability to inhibit COX-2 enzyme compared with COX-1, which suggests the antiarthritic potential of *S. sesuvioides*.

## 4 Materials and methods

### 4.1 Plant material and preparation of extract

Plants were collected in the Rohi area (N: 28^o^49.208′ E: 071^o^28.129′, at the elevation of 334 ft, interdunal sand) of the Cholistan Desert in Pakistan (CDP). The plant was authenticated and deposited in the herbarium of the Cholistan Institute of Desert Studies with voucher no. CIDS/IUB-0206/08 by Dr. Muhammad Abdullah, a plant taxonomist at The Islamia University of Bahawalpur, Pakistan. The whole plant was shade-dried, crushed, and extracted with 70% (v/v) aqueous CH_3_OH under reflux. The collected eluent was dried using a rotary evaporator under a vacuum (Rotavapor R-200 Buchi, Germany) at 37°C. After drying, it was further partitioned with *n*-butanol:water (1:1 v/v). The *n*-butanol fraction was then concentrated until dry and was abbreviated to SsBu for future analysis (Prashant et al., 2011).

### 4.2 Drugs and chemicals

Formalin (Riedel-de Haen, Germany), complete Freund’s adjuvant (Sigma-Aldrich, United States), diclofenac sodium (Sami Pharmaceuticals), bovine serum albumin (5% w/v) (Sigma-Aldrich, United States), fresh egg albumin, EDTA (10%), phosphate-buffered saline (pH = 6.4), plethysmometer, ELISA kits of superoxide dismutase (SOD) (Cat No. *E-BC-K-020*, Elabscience^®^), gultathion reductase (GSH) (Cat No. *E-EL-R-2491*, Elabscience^®^), malondialdehyde (MDA) (Cat No. *E-EL-0060*, Elabscience^®^), interleukin-6 (Cat No. *E-EL-H-0109*, Elabscience^®^), TNF-α (Cat No. *E-EL-R-2491*, Elabscience^®^), and PGE-2 (Cat No. *E-EL-R-0034*, Elabscience^®^) were used. SsBu_1_ (250 mg/kg), SsBu_2_ (500 mg/kg), and SsBu_3_ (750 mg/kg) were also used.

### 4.3 Preliminary phytochemical analysis

The presence of different secondary metabolites and total phenolic and flavonoid contents were performed and reported as described previously (Sajid-ur-Rehman et al., 2021).

### 4.4 Determination of total phenolic contents

Total phenolic contents of the butanol fraction (SsBu) were measured using the modified colorimetric Folin–Ciocalteu reagent (FCR) method, and data were expressed as milligram gallic acid equivalent per gram of dry extract (mg of GAE/g extract) with triplicate assessments. A measure of 10 µl of FCR was mixed with 100 µl of sample solution (SS) and incubated for 10 min, and then 90 µl of (15% W/V) sodium carbonate aqueous solution was added. This mixture was incubated for 90 min at 37 °C, and the absorbance at 750 nm was measured.

### 4.5 Determination of total flavonoid contents

Total flavonoid contents were assessed using an AlCl_3_ colorimetric assay with slight modification (Khan et al., 2019). A measure of 100 μl of each SS, 25 Iµl (1%) of sodium nitrite solution, and 10 µl (10%) of AlCl_3_ solution was mixed and allowed to react for 5 min. Finally, 35 µl of 4% sodium hydroxide solution was added, and the mixture was diluted with 30 µl of methanol. The absorbance at 510 nm was measured. TFCs were calculated using the calibration curve equation and expressed as mg rutin equivalent per gm of dry extract (mg of RE/g extract).

### 4.6 Antioxidant assays

Antioxidant activities, including DPPH, ABTS, FRAP, CUPRAC, phosphomolybdenum, and metal chelating activity, were assessed according to protocols described previously (Saleem et al., 2021; Khursheed et al., 2022).

### 4.7 GC-MS analysis

GC-MS analysis of SsBu was performed using the GC-B-7890 system furnished with a mass detector (MSD-5977IA). Phytocompounds were separated using a column (HP-5MS, 30 × 0.25 × 0.25) having 100% dimethyl polysiloxane as the stationary phase and 2 µl of the sample as the mobile phase at 250°C. The carrier gas was helium with a flow rate of 1.0 ml/min (Gomathi et al., 2015).

### 4.8 Determination of the prostaglandin E-2 level

In this assay, 100 µl of sample, standard, and blank solutions and 100 µl of PGE-2 antibody solution were placed in the wells of the ELISA plate and incubated at 37°C for 60 min. Then, ELISA plates were covered and placed in a shaker incubator at 500 rpm for 2 h. The wells were decanted and washed three times with washing buffer solution. Also, 100 µl of PGE-2 alkaline phosphate and 90 µl *p*-nitrophenyl phosphate substrate reagent were added to all wells and incubated at 37°C for 15 min. A volume of 50 µl of stop solution was added to terminate the reaction, and the OD value at 450 nm was determined using a microplate reader (Jabeen et al., 2022).

### 4.9 *In vitro* and *in vivo* antiarthritis activity

#### 4.9.1 Inhibition of protein denaturation using egg albumin

For this assay, a reaction mixture containing 0.2 ml of fresh hen egg albumin, 2.8 ml of phosphate-buffered saline, and 2 ml of SsBu, along with diclofenac sodium in various concentrations (25, 50,100, 200, 400, and 800 μg/ml), was prepared. The mixtures were incubated at 37°C for 15 min, and the absorbance at 660 nm was measured (Khalid et al., 2021; Qasim et al., 2021). The inhibition (%) of protein denaturation was calculated using the following formula:

$$Inhibition\;\left(\%\right)=100\times \frac{Absorbance\;of\;Test\;Sample}{Absorbance\;of\;Control}-1$$ (1)

#### 4.9.2 Inhibition of protein denaturation using bovine serum albumin

For this assay, a reaction mixture containing 5% bovine serum albumin and 0.05 ml of SsBu, in various concentrations (25, 50, 100, 200, 400, and 800 μg/ml), along with aspirin (standard drug), was prepared and incubated at 37°C for 20 min. Then, 2.5 ml of phosphate-buffered saline (pH = 6.3) was added, and the absorbance at 660 nm was measured (Qasim et al., 2021). The inhibition (%) of protein denaturation was calculated using the following formula:

$$Inhibition\;\left(\%\right)=100-\frac{Absorbance\;of\;Sample-Absorbance\;of\;Product\;Control}{Absorbance\;of\;Test\;Control}\times 100.$$ (2)

#### 4.9.3 Formaldehyde-induced arthritis

In this assay, Wistar albino rats (150–200 g) were divided into six groups (n = 6). Formaldehyde (0.1 ml 5% V/V) was injected (days 1 and 3) into the left hind paw of experimental rats except for the normal control group. The treatment (SsBu_1_, SsBu_2_, and SsBu_3_) was provided for 10 days, and the thickness of the rat’s paw was assessed (Saleem et al., 2019).

#### 4.9.4 Complete Freund’s adjuvant (CFA)-induced arthritis

Male albino rats (150–200 g) were divided into six groups (n = 6). All animals were acclimatized to the laboratory environment for a week, and 0.1 ml of CFA was injected subcutaneously into the left hind paw of all animals except the control group. The control group and intoxicated group (arthritic group) received 5 ml/kg of 10% DMSO. The standard group received diclofenac sodium (5 mg/kg), while the treatment group received SsBu_1_, SsBu_2,_ and SsBu_3,_ and the dosing was continued until day 28. Animal paw edema and body weight changes were assessed on days 7, 14, 21, and 28 *via* the water displacement method using a plethysmometer (Ugo Basile, Italy). On day 29, the animals were anesthetized, and blood was collected from the retro-orbital cavity to estimate the hematological parameters, biochemical oxidative stress markers, including SOD, MDA, and GSH, and pro-inflammatory mediators, including IL-6 and TNF-α, by the ELISA kit method. Finally, all rats were sacrificed by a painless procedure of cervical dislocation, and left hind paws were removed and secured in 10% formalin. The left hind paws were decalcified by EDTA (10%) for a period of 30 days and implanted in paraffin wax. The paw tissues were then sliced (5 µm) using a microtome, stained, and viewed for histopathological changes under a microscope (Amin et al., 2022).

#### 4.9.5 Ethical statement

The experimental procedure was performed under the NIH (National Institutes of Health, Publication No. 85–23) guidelines regarding animal ethics and reviewed by the Pharmacy Animal Ethics Committee with Reference No. PAEC 20/30 issued by the Faculty of Pharmacy, IUB Punjab, Pakistan.

### 4.10 Estimation of oxidative stress markers

The levels of oxidative markers were determined by estimating the concentrations of SOD, GSH, and MDA according to the procedure provided in the ELISA kits.

#### 4.10.1 Estimation of SOD

In this procedure, control solution (20 µl of ddH_2_O), working solution (200 µl of substrate solution), sample solution (20 µl of working solution, a plasma sample, and 200 µl of the substrate solution), blank sample solution (20 µl of ddH_2_O, enzyme diluent and 200 µl of substrate application solution), and blank kit solution (20 µl of sample, 20 µl of enzyme diluent, and 200 µl of the substrate solution) were added separately. All the solutions were mixed thoroughly and incubated at 37°C for 20 min, and the OD values at 450 nm were measured (Lodhi et al., 2021).

#### 4.10.2 Estimation of GSH

In this assay, 100 µl of standard working solution, SS (sample solution), and biotinylated Ab working solution (100 µl) were added to each well of the ELISA plate and incubated at 37°C for 90 min. The wells were rinsed with buffer solution (350 µl), and 100 µl of HRP conjugate working solution was added and incubated at 37°C for 30 min. Again, each well was rinsed, and 90 µl of substrate reagent was added. A measure of 50 µl of stop solution was added to terminate the reaction. The OD values at 450 nm were determined using a microplate reader (Lodhi et al., 2021).

#### 4.10.3 Estimation of MDA

In this assay, 50 µl of standard working solution, SS, and antibody working solution (50 µl) were added to each well of the ELISA plate and incubated at 37°C for 45 min. The wells were rinsed with buffer solution (350 µl), and 100 µl of HRP conjugate working solution was added and incubated at 37°C for 30 min. Finally, 90 µl of substrate reagent and 50 µl of stop solution were added, and the OD values at 450 nm were determined using a microplate reader (Lodhi et al., 2021).

### 4.11 Estimation of levels of pro-inflammatory markers

#### 4.11.1 Estimation of the level of IL-6

In this assay, 100 µl of SS and 100 µl of biotinylated detection antibody solution were added to each well of the ELISA plate and incubated at 37°C for 60 min. The wells were rinsed with buffer solution (350 µl), and 100 µL of HRP conjugate solution was added and incubated at 37°C for 30 min. Finally, the wells were rinsed with buffer solution, and 90 µl of substrate reagent and 50 µl of stop solution were added to each well. The OD value at 450 nm was determined using a microplate reader (Javed and Jabeen, 2021).

#### 4.11.2 Estimation of levels of TNF-α

In this assay, 100 µl of the sample, standard, and blank solutions and 100 µl of biotinylated detection antibody solution were added to wells of the ELISA plate and incubated at 37°C for 90 min. The wells were rinsed with buffer solution (350 µl), and 100 µl of HRP conjugate solution was added to the wells and incubated at 37°C for 30 min. Finally, the wells were rinsed with wash buffer, and 90 µl of substrate reagent and 50 µl of stop solution were added. The OD value at 450 nm was determined using a microplate reader (Javed and Jabeen, 2021)./

### 4.12 Molecular docking studies

#### 4.12.1 Preparation of enzyme and ligand molecules

Structures of cyclooxygenase-1 (6y3c) and cyclooxygenase-2 (6bl4), with the highest resolution, were obtained from the Protein Data Bank in the PDB format. The Discovery Studio (DS 2021 Client) program was used to prepare protein molecules. Attached molecules, including water molecules and other ligand structures, were removed from macromolecules. Afterwards, they were transferred onto the PyRx program for docking purposes in a PDBQT file format that contains a protein structure with hydrogens in all polar residues. Major compounds identified by GC-MS were selected as ligands, and their structures were obtained from the NIST library and Pubchem in 3D SDF formats. The software specification and procedure of docking were followed as described by Ahmed et al. (2022a).

#### 4.12.2 Docking interaction of phytochemicals and macromolecules

The molecular docking was performed using PyRx version 0.8 software. The enzyme macromolecule was loaded into PyRx, and “Make Macromolecule” was used to define the protein and ligand using AutoDock embedded in PyRx. Then, the ligands were added in the Open Babel tool, and energy was minimized to obtain the stable conformation; then, the molecules were converted to the PDBQT format in AutoDock. The docking site on the protein target was defined by establishing a grid box with the dimensions of X: 28.0238, Y: 40.4164, and Z: 22.3236 A^о^ for COX-1 and X: 47.7267, Y: 25.5073, and Z: 20.7618 A^о^ for COX-2, with a grid box size of 50 × 50 × 50 A^о^, and exhaustiveness was 8. The other settings of the software were used as “default.” The best conformation with the lowest docked energy was chosen after the docking search was completed. The docking was repeated with 10 runs for each enzyme and ligand, the best pose was saved, and the average affinity for the best poses was taken as the final affinity value in terms of binding energy. The molecular docking result for each compound was visualized as an output PDBQT file by using the molecular graphics laboratory (mgltools) tool. PyRx software is an easy, non-commercial, and less time-consuming virtual tool that basically predicts receptor–ligand binding and provides energy values for each test compound. The 2D and 3D interaction of the individual phytochemical against COX-1 (Figure 12) and COX-2 (Figure 13) proteases was finally visualized in Discovery Studio using mgltools, to determine some specific contacts between the atoms of the test compounds and amino acids of the studied receptor.

### 4.13 Statistical analysis

Results are presented as mean ± SEM (*n* = 6). One-way ANOVA was applied, followed by Bonferroni’s test using GraphPad Prism (San Diego, CA, United States) software, and *p* < 0.05 was considered statistically significant.

## 6 Conclusion

From our findings, we concluded that SsBu effectively inhibited protein denaturation, decreased the hind paw volume, and improved the hematological parameters in arthritic animals. The significant antiarthritic activity could be attributed to its ability to suppress levels of oxidative stress markers and pro-inflammatory cytokines. Molecular docking of major identified compounds showed the COX-1 and COX-2 inhibitory potential of SsBu. Collectively, these findings demonstrated the pharmacological potential of SsBu in treating RA.

## Data Availability

The original contributions presented in the study are included in the article/Supplementary Materials; further inquiries can be directed to the corresponding authors.
